# Survival among people hospitalized with COVID-19 in Switzerland: a nationwide population-based analysis

**DOI:** 10.1186/s12916-022-02364-7

**Published:** 2022-04-26

**Authors:** Nanina Anderegg, Radoslaw Panczak, Matthias Egger, Nicola Low, Julien Riou

**Affiliations:** 1 Institute of Social and Preventive Medicine, University of Bern, Bern, Switzerland; 2 Federal Office of Public Health, Bern, Switzerland; 3 Centre for Infectious Disease Epidemiology and Research, Faculty of Health Sciences, University of Cape Town, Cape Town, South Africa; 4 Population Health Sciences, Bristol Medical School, University of Bristol, Bristol, UK

**Keywords:** COVID-19, Survival, SARS-CoV-2, Intensive care unit

## Abstract

**Background:**

Increasing age, male sex, and pre-existing comorbidities are associated with lower survival from SARS-CoV-2 infection. The interplay between different comorbidities, age, and sex is not fully understood, and it remains unclear if survival decreases linearly with higher ICU occupancy or if there is a threshold beyond which survival falls.

**Method:**

This national population-based study included 22,648 people who tested positive for SARS-CoV-2 infection and were hospitalized in Switzerland between February 24, 2020, and March 01, 2021. Bayesian survival models were used to estimate survival after positive SARS-CoV-2 test among people hospitalized with COVID-19 by epidemic wave, age, sex, comorbidities, and ICU occupancy. Two-way interactions between age, sex, and comorbidities were included to assess the differential risk of death across strata. ICU occupancy was modeled using restricted cubic splines to allow for a non-linear association with survival.

**Results:**

Of 22,648 people hospitalized with COVID-19, 4785 (21.1%) died. The survival was lower during the first epidemic wave than in the second (predicted survival at 40 days after positive test 76.1 versus 80.5%). During the second epidemic wave, occupancy among all available ICU beds in Switzerland varied between 51.7 and 78.8%. The estimated survival was stable at approximately 81.5% when ICU occupancy was below 70%, but worse when ICU occupancy exceeded this threshold (survival at 80% ICU occupancy: 78.2%; 95% credible interval [CrI] 76.1 to 80.1%). Periods with higher ICU occupancy (>70 vs 70%) were associated with an estimated number of 137 (95% CrI 27 to 242) excess deaths. Comorbid conditions reduced survival more in younger people than in older people. Among comorbid conditions, hypertension and obesity were not associated with poorer survival. Hypertension appeared to decrease survival in combination with cardiovascular disease.

**Conclusions:**

Survival after hospitalization with COVID-19 has improved over time, consistent with improved management of severe COVID-19. The decreased survival above 70% national ICU occupancy supports the need to introduce measures for prevention and control of SARS-CoV-2 transmission in the population well before ICUs are full.

**Supplementary Information:**

The online version contains supplementary material available at 10.1186/s12916-022-02364-7.

## Background

More than five million people have died from coronavirus disease 19 (COVID-19), and almost 250 million confirmed cases of severe acute respiratory syndrome coronavirus 2 (SARS-CoV-2), the cause of COVID-19, have been reported worldwide as of November 08, 2021 [1–3]. The risk of death from SARS-CoV-2 infection increases with age and is higher for men than women [4–12]. In addition, people with pre-existing comorbid conditions, including cancer, cardiovascular disease, and chronic respiratory disease, are at higher risk of severe COVID-19 or death than people without these conditions [7, 8, 13–18]. The interplay between different comorbid conditions and their associated risk of death for different ages or genders is not fully understood.

In many countries, strict lockdown measures controlled the first wave of SARS-CoV-2 infection in the first half of 2020, but a second larger wave followed the delayed re-introduction of control measures in autumn 2020. Switzerland, a high-income country, experienced the highest levels of excess mortality since the 1918 influenza pandemic, with a larger death toll during the second compared to the first epidemic wave [19, 20]. As in many other countries, the number of available intensive care unit (ICU) beds has become an indicator of the need to introduce or intensify public health measures to control SARS-CoV-2 transmission. High ICU occupancy during peaks of the epidemic has been reported to lead to worse outcomes in people who are hospitalized with COVID-19, possibly due to operational pressure [21, 22]. It is unknown to what extent this applies to Switzerland. In addition, it remains unclear if outcomes decrease linearly with higher ICU occupancy or if there is a threshold beyond which survival falls.

The objectives of this study were to examine differences in survival between the first and second epidemic wave in Switzerland, the influence of the level of ICU occupancy on survival, and the interplay between different comorbid conditions and other risk factors.

## Methods

### Setting and data

We analyzed data for the whole of Switzerland from national routine surveillance of SARS-CoV-2 infections, conducted by the Swiss Federal Office of Public Health. The surveillance system includes all people who tested positive for SARS-CoV-2 infection in Switzerland since February 24, 2020, when the first case of COVID-19 was diagnosed. Laboratories report the date of the positive test and the age and sex of the person. For patients hospitalized, medical personnel report the date of hospitalization and information about specific comorbid conditions. For patients who die, medical personnel report the date of death and the presence of comorbid conditions. For the present analysis, we included all patients with a positive test for SARS-CoV-2 infection who were hospitalized between February 24, 2020, and March 01, 2021, and who had complete information about age, sex, and comorbid conditions. The database was closed on May 20, 2021, so that all patients were followed up for at least 80 days after the positive test.

The Swiss Armed Forces delivered daily aggregated data from all hospitals in Switzerland on the total number of available ICU beds and of occupied beds since March 14, 2020. The total occupied includes beds occupied by patients with COVID-19 and without COVID-19. The total available beds consist of a relatively stable number of ICU beds over the whole period (“certified ICU beds”) and a variable number of additional ICU beds, which have been made available, depending on the state of the epidemic in the country. These are called “add-on” ICU beds.

### Outcomes

The outcome was survival after a positive test for SARS-CoV-2 infection in patients hospitalized with COVID-19. We measured follow-up time from the positive SARS-CoV-2 test to the date of death for patients who died by May 20, 2021. We assumed that deaths occurring more than 80 days after infection were not attributable to COVID-19. The date of hospital discharge was not available, as the surveillance system does not require a follow-up report after hospital admission. Therefore, we censored follow-up time at 80 days after the positive test for people who died more than 80 days after a positive test and for all people alive at database closure.

### Explanatory variables

Explanatory variables were the epidemic wave, sex and age, comorbidities, and ICU occupancy. We defined the first epidemic wave as the period between February 24, 2020, and June 5, 2020. The second wave covered June 6, 2020, to March 01, 2021. The date separating the two waves was the nadir of the case counts between the two waves. We defined daily ICU occupancy as the proportion of all occupied ICU beds (COVID-19 and non-COVID-19) in Switzerland on a given day divided by the total number of available ICU beds (certified and add-on beds) in Switzerland on the same day. Age was grouped into eight categories, 0–29, 30–39, 40–49, 50–59, 60–69, 70–79, 80–89, and ≥90 years. We included all comorbid conditions that were recorded on the surveillance form. These were cancer, cardiovascular disease, chronic respiratory disease, diabetes, hypertension, immunosuppression, obesity, chronic kidney disease, “other” comorbidity, and no comorbidity. Obesity and chronic kidney disease were added to the surveillance forms from April 16, 2020.

### Statistical analyses

We modeled the hazard of death by fitting parametric survival models in a Bayesian framework. We used cubic M-splines to model baseline hazards for all survival models in this study and assumed weakly informative priors for the model parameters (Additional file 1: Text S1) [23]. To compare survival between the two epidemic waves, we used data for the whole study period and fitted a survival model, including the covariates epidemic wave, age, sex, and a binary variable indicating any or no recorded comorbid condition. We also included all two-way interactions between covariates age, sex, and comorbidity status. We fitted the same model in an additional analysis but included the number of comorbid conditions reported (0, 1, 2, or 3 or more). We used these results to build models to study survival by ICU occupancy and by type of comorbid condition. More specifically, we assessed the evidence for the different two-way interactions by comparing the fit of the full model including all two-way interactions between age, sex, and comorbidity status to alternative models including only a subset of these interactions. Model fits were compared by looking at their difference in expected log pointwise predictive density (and its standard error) for a new dataset, estimated by approximate leave-one-out cross-validation [23].

For the models studying survival by ICU occupancy and by type of comorbid condition, we only used data from the second wave because data on ICU occupancy, obesity, and chronic kidney disease during the first wave were incomplete. For ICU occupancy at the national level, we modeled the association with survival among patients who were hospitalized with COVID-19 using restricted cubic splines with three knots (at the 10%, 50%, and 90% percentiles of the ICU occupancy distribution in our data) (Additional file 1: Text S1). We adjusted the model for age, sex, and comorbidity status. We also included the two-way interaction between age and comorbidity status, the only interaction for which we found strong evidence in the first model (Additional file 1: Text S1). To investigate survival by type of comorbidity, we included the covariates age and sex and the different comorbidity types. We allowed for two-way interactions between the different comorbidity types, between age and the comorbidity types, and between sex and the comorbidity types (Additional file 1: Text S1).

We visually checked for strong deviations from the proportional hazards assumption by plotting log(-log(S(t))) versus time separately for each covariate included in the survival models. We checked model fits visually by comparing the estimated marginal survival function based on draws from the posterior predictive distribution of the fitted joint model to the Kaplan-Meier curve based on the observed data.

We report estimates of adjusted hazard ratios (aHR) for death, predicted survival curves over time (i.e., predicted survival probability at day 0 to day 80 after the positive test), and—as a specific example—the predicted survival probability at day 40 after the positive test, all with 95% credible intervals (CrI). We also report standardized survival probabilities by specific covariates (epidemic wave and ICU occupancy). We standardized by predicting individual survival probabilities for the whole population at all levels of the specific covariate(s) and averaging predictions in each of the posterior samples. All analyses were done using R (version 4.0.4) with package *rstanarm* [23].

## Results

### Descriptive statistics

**Fig. 1:**
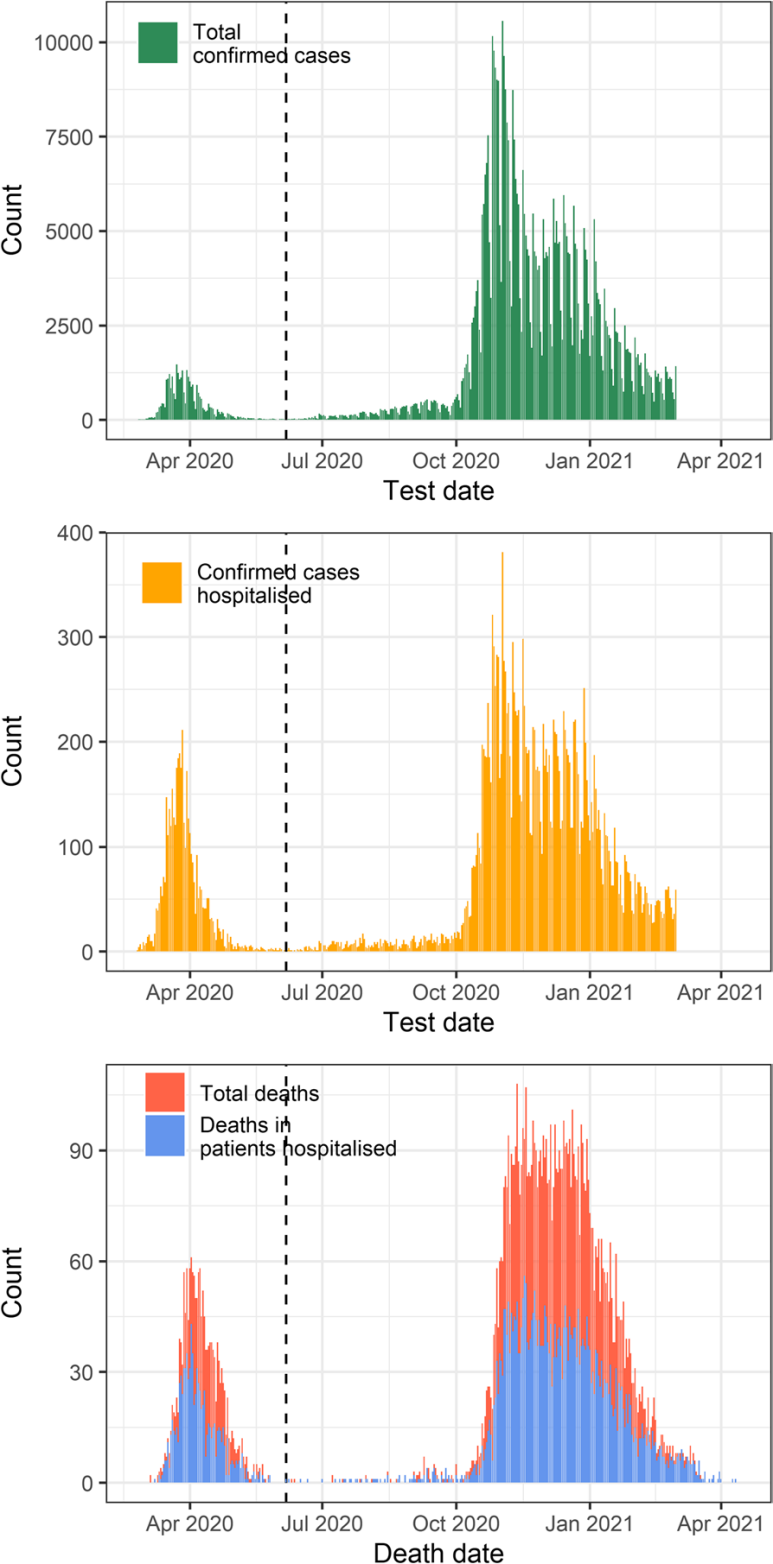
The SARS-CoV-2 epidemic in Switzerland. Total confirmed cases from February 24, 2020, to March 01, 2021 (upper panel), confirmed hospitalized patients (middle panel), and total deaths and deaths in patients hospitalized until May 20, 2021 (lower panel). The dashed vertical lines correspond to the end of the first epidemic wave

Overall, there were 559,117 confirmed COVID-19 cases between February 24, 2020, and March 01, 2021, of whom 24,515 (4.4%) were hospitalized (Fig. 1). Of all people with confirmed COVID-19, 9711 (17%) had died by May 20, 2021. Of patients hospitalized, 4847 (19.8%) had died, covering 49.9% of the total reported deaths. People who died outside of the hospital include retirement and nursing homes residents, but information about the place of death was incomplete. People older than 90 years accounted for 42.1% (1949/4630) of deaths outside the hospital and for 15.6% (746/4785) of in-hospital deaths (Additional file 1: Table S1).

We included 22,648/24,515 (92.4%) patients who were hospitalized with COVID-19 and had complete covariate information in our analyses (Additional file 1: Fig. S1). Of the excluded 1867 (7.6%) patients, most (1864) were excluded due to missing information about comorbid conditions. Patient characteristics of those with and without missing comorbid conditions were similar, but mortality was lower in those with missing comorbid information (Additional file 1: Table S2). Of all included patients, 4785 (21.1%) had died (Table 1). The median (interquartile range [IQR]) age of patients was 74 (62 to 83) years, and most were male (13,093, 57.8%). Most patients had at least one comorbid condition (19,865, 87.7%). More people diagnosed with SARS-CoV-2 were hospitalized in the second epidemic wave (18,952) than in the first (3696). Still, the proportion of people who died among those hospitalized was higher in the first (24.3%) than in the second wave (20.5%). Sex and age distributions were similar in the two epidemic waves, as was the presence of specific comorbid conditions (except for obesity and chronic kidney disease, which were probably mostly recorded as “other” comorbidity in the first wave). During the second wave, the total number of available ICU beds ranged between 963 and 1143, consisting of 781 to 861 certified beds and 135 to 296 add-on beds (Additional file 1: Fig. S2). ICU occupancy among all available beds ranged between 51.7 and 78.8%. In periods with low ICU occupancy (1st decile, ≤65.7% occupancy) 327 of 1903 (17.2%) patients hospitalized with COVID-19 died, while in periods with high ICU occupancy (10th decile, >76.9% occupancy) 431 of 1890 (22.8%) patients died (Table 1).

**Table 1:** Characteristics and mortality of patients hospitalized with a positive SARS-CoV-2 test during the first and second epidemic wave in Switzerland

	First epidemic wave February 24 to June 5, 2020			Second epidemic wave June 6, 2020, to March 1, 2021		
	No. of patients	Deaths	%	No. of patients	Deaths	%
Total	3696	897	24.3%	18,952	3888	20.5%
**Sex**						
Female	1456	292	20.1%	8099	1381	17.1%
Male	2240	605	27.0%	10,853	2507	23.1%
**Age [years]**						
Median (IQR)	73 (59–82)	81 (74–87)		75 (63–83)	82 (75–87)	
0–29	137	1	0.7%	517	3	0.6%
30–39	122	4	3.3%	399	4	1.0%
40–49	202	4	2.0%	874	22	2.5%
50–59	505	31	6.1%	2009	98	4.9%
60–69	624	94	15.1%	3274	382	11.7%
70–79	920	252	27.4%	4976	1107	22.2%
80–89	906	371	40.9%	5360	1666	31.1%
90+	280	140	50.0%	1543	606	39.3%
**Comorbid conditions**						
**Presence of any condition**						
No comorbid condition	495	19	3.8%	2288	88	3.8%
Any comorbid condition	3201	878	27.4%	16,664	3800	22.8%
**Number of conditions**						
0	495	19	3.8%	2288	88	3.8%
1	1184	149	12.6%	5253	569	10.8%
2	1006	254	25.2%	4809	901	18.7%
3+	1011	475	47.0%	6602	2330	35.3%
**Presence of specific condition**						
Cancer	382	182	47.6%	2203	795	36.1%
Cardiovascular disease	1244	534	42.9%	8057	2561	31.8%
Chronic kidney disease	90	61	67.8%	3803	1437	37.8%
Chronic respiratory disease	578	209	36.2%	3135	960	30.6%
Diabetes	844	258	30.6%	4875	1274	26.1%
Hypertension	1921	602	31.3%	9781	2551	26.1%
Immunosuppression	153	63	41.2%	1036	322	31.1%
Obesity	16	8	50.0%	1844	430	23.3%
Other	1500	481	32.1%	4657	1254	26.9%
**ICU occupancy**						
**Total ICU occupancy**						
≤ 65.7%^a^	-	-		1903	327	17.2%
65.8–72.8%^a^	-	-		7622	1521	20.0%
72.9–76.9%^a^	-	-		7537	1609	21.3%
> 76.9%^a^	-	-		1890	431	22.8%

^a^65.7, 72.8, and 76.9% correspond to the 10%, 50%, and 90% percentiles of the ICU occupancy distribution in the second epidemic wave. Abbreviations: *ICU* intensive care unit. Figures correspond to counts (percentage) or median (interquartile range)

### Survival by epidemic wave

**Fig. 2:**
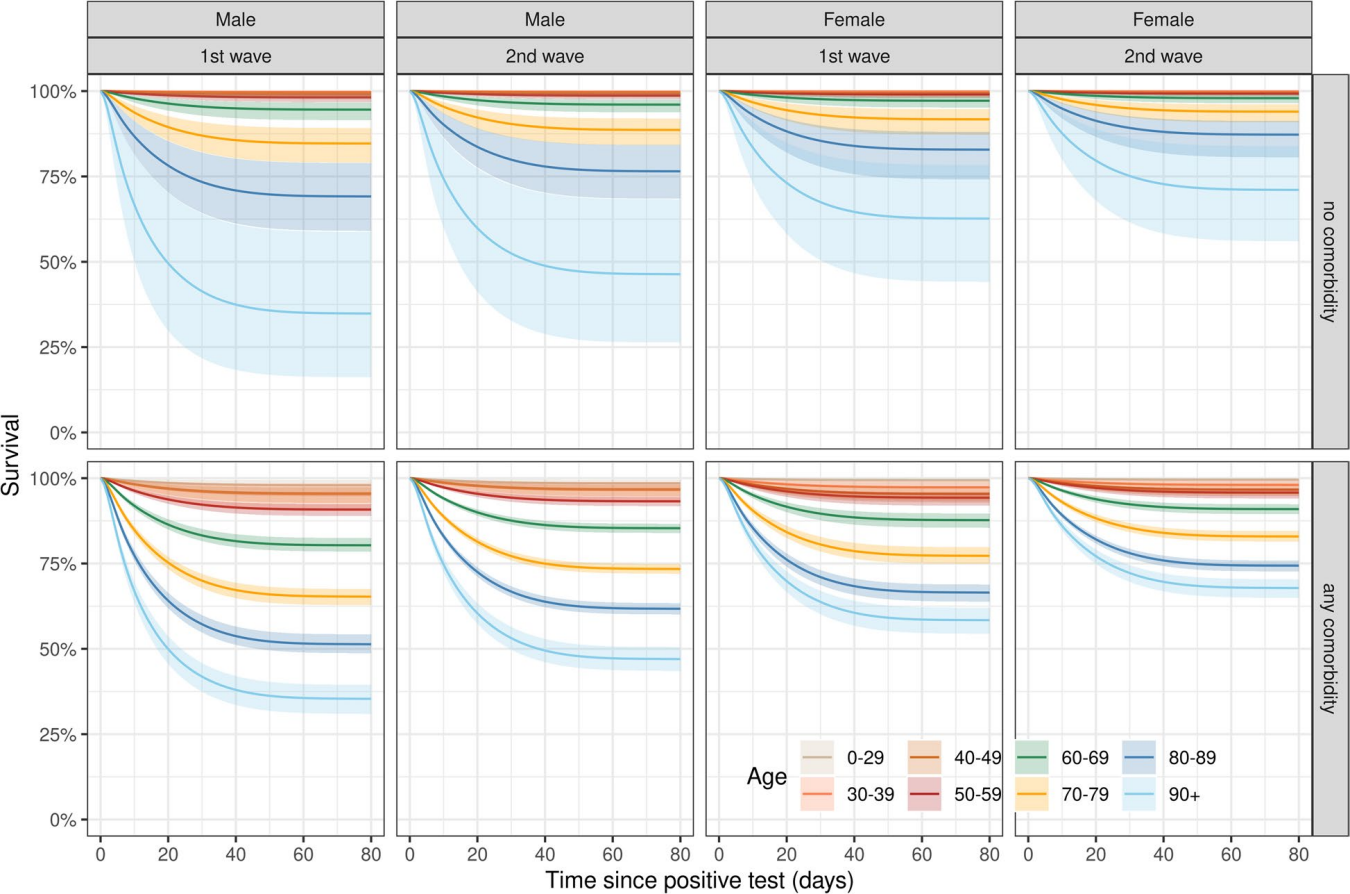
Survival among hospitalized COVID-19 patients by epidemic wave, sex, age, and comorbidity status. Survival curves and 95% credible intervals over time after the date of the positive SARS-CoV-2 test. Results from the model fitted to data from both epidemic waves including covariates epidemic wave, sex, age, comorbidity status, and all two-way interactions between age, sex, and comorbidity status

**Fig. 3:**
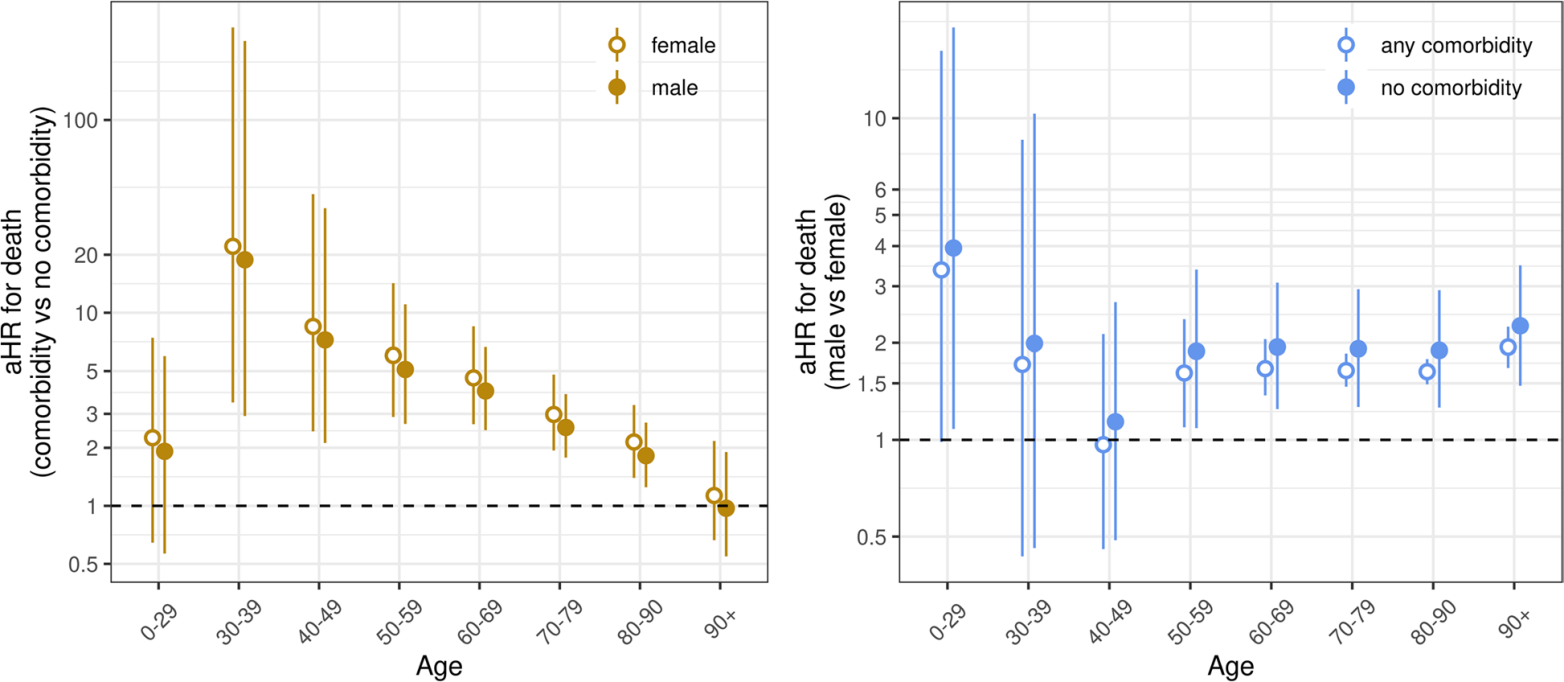
Estimated adjusted hazard ratios (aHR) and 95% credible intervals (CrI) for two-way interactions between variables sex, age, and comorbidity status. The left panel (brown) compares the hazard of death for hospitalized COVID-19 patients with comorbidity to those without comorbidity by age group. The right panel (blue) compares the hazard of death for males to females by age group. Results from the model fitted to data from both epidemic waves including covariates epidemic wave, sex, age, comorbidity status, and all two-way interactions between age, sex, and comorbidity status

The baseline risk of death increased sharply after the positive SARS-CoV-2 test, peaked at around 4.5 days, and then decreased again, approaching zero at around day 70 (Additional file 1: Fig. S3). There was a substantial increase in the hazard of death during the first epidemic wave compared with the second wave, after adjusting for age, sex, and the presence of any comorbid condition (aHR 1.38, 95% CrI 1.28 to 1.48). The standardized predicted probability of survival at 40 days was 76.1% (95% CrI 75.1 to 77.2%) during the first and 80.5% (95% CrI 80.0 to 81.1%) during the second epidemic wave (Additional file 1: Fig. S3). Mortality also increased with male sex, older age, and with the presence of any comorbidity (Fig. 2). There was evidence for an interaction between comorbidity and age: the presence of comorbidity increased the risk of death more at younger ages than at older ages (Fig. 3, Additional file 1: Text S1). For example, in men, the aHR of death with comorbidity compared with no comorbidity was 3.94 (95% CrI 2.47 to 6.64) for ages 60–69 years and only 0.97 (95% CrI 0.55 to 1.90) for ages above 90 years. There was no evidence of an interaction between sex and comorbidity status (Fig. 3, Additional file 1: Text S1). There was also little evidence of an interaction between sex and age. The difference in mortality by sex in the age groups 0–49 years was unclear owing to small sample sizes, but for patients aged above 50 years, there was a consistent increase in the hazard of death by around 1.6 to 1.7 in males compared with females (Fig. 3, Additional file 1: Text S1). The additional analysis, including the number of comorbidities instead of the comorbidity status, showed that mortality generally increased with the number of reported comorbidities (Additional file 1: Fig. S4). Again, there was a substantial interaction between comorbidity and age group.

### Survival by ICU occupancy

**Fig. 4:**
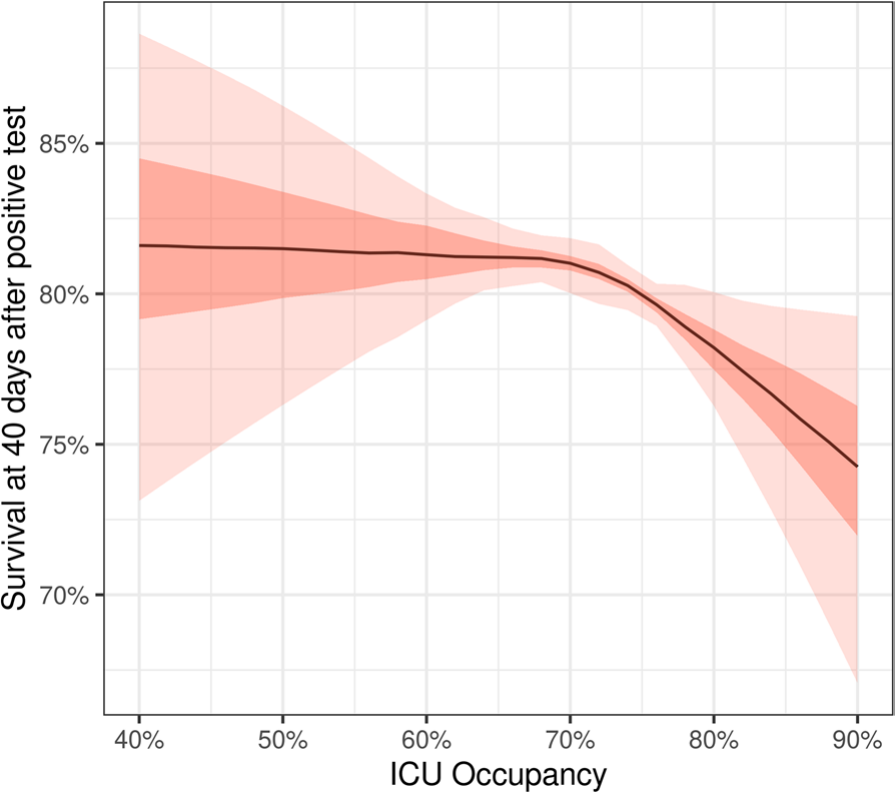
Survival of hospitalized COVID-19 patients by ICU occupancy. Standardized predicted survival at 40 days after positive test by ICU occupancy. More transparent areas correspond to 95% credible intervals and less transparent ones to 50% credible intervals. Results from the model fitted to data from the second epidemic wave, including covariates ICU occupancy (modeled by restricted cubic splines with 3 knots), sex, age, comorbidity status, and the interaction between age and comorbidity status. Standardization is done by averaging predictions at 40 days for the whole population by ICU occupancy in each of the posterior samples (1000 draws)

The hazard of death was relatively stable up to around 70% ICU occupancy and started to increase afterwards (Additional file 1: Fig. S5). Standardized predicted survival at 40 days was stable up to 70% ICU occupancy but decreased at higher levels of occupancy (Fig. 4). For example, estimated standardized survival at 40 days was similar at 50% ICU occupancy (81.5%; 95% CrI 76.3 to 86.2%) and 70% ICU occupancy (81.0%; 95% CrI 80.0 to 81.9%). Survival at 40 days started to decrease from this point on, reaching 78.2% (95% CrI 76.1 to 80.1%) at 80% ICU occupancy. Focusing on periods in which ICU occupancy exceeded 70%, these results correspond to an estimated number of 137 (95% CrI 27 to 242) deaths—or an estimated proportion of 4.80% (95% CrI 0.94 to 8.29%) of total deaths during these periods—that could have been averted if ICU occupancy had stayed at 70% during these periods. The 70% ICU occupancy threshold among all available beds corresponded to approximately 85% occupancy among certified beds during the second epidemic wave (Additional file 1: Fig. S2). Associations between mortality and covariates sex, age, and comorbidity status obtained were similar to those observed with the model comparing the epidemic waves.

### Survival by comorbidity type

**Fig. 5:**
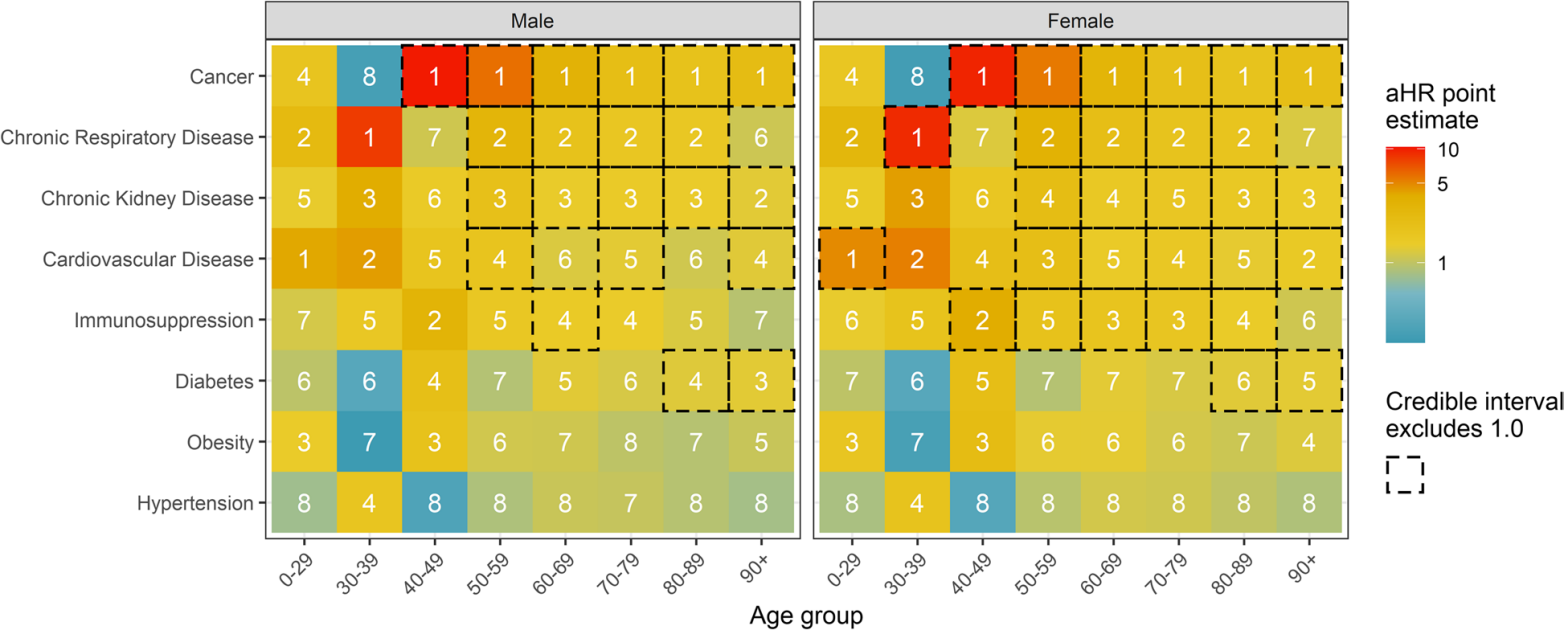
Heat map of adjusted hazard ratios (aHR) of death comparing hospitalized COVID-19 patients with a single comorbid condition to patients free of comorbidity. Boxes with dashed frames indicate 95% credible intervals (CrI) excluding 1. White numbers correspond to ranks of the importance of comorbidities based on the aHR. Rank 1 corresponds to the comorbidity with the strongest association with mortality. Results from the model fitted to data from the second epidemic wave, including covariates sex, age, the different comorbidity types, and two-way interactions between comorbidity types, between age and comorbidity types, and between sex and comorbidity types

In patients below the age of 40 years, we found insufficient and inconclusive evidence for any associations between single comorbid conditions and mortality. The small number of deaths in these age groups leads to imprecise estimates with wide credibility intervals (Fig. 5). In patients aged 40 years or older with a single comorbid condition, the strongest association with mortality was cancer. For patients aged 50–89 years old, chronic respiratory disease, chronic kidney disease, cardiovascular disease, and immunosuppression were also often associated with higher mortality. There was little evidence for worse survival in patients with hypertension or obesity as the only comorbid condition. The patterns of association were similar for males and females. Looking at two-way interactions between comorbidity types, we found that having both hypertension and cardiovascular disease was more strongly associated with death than the sum of the individual effects of both (Additional file 1: Fig. S6). This suggests a synergistic effect of these two conditions. Other potential synergies include obesity and diabetes, immunosuppression and chronic kidney disease, or obesity and hypertension. We also found some indication that the combination of obesity and cancer or immunosuppression, or diabetes and chronic respiratory disease was less strongly associated with mortality than the sum of the individual conditions (antagonist effect). However, for most of these combinations, effect sizes were small and credible intervals overlapped 1—indicating weak evidence of an interaction.

### Model diagnostics

For most covariates, plotting of log(-log(S(t))) versus time resulted in parallel lines indicating no strong deviation from the proportional hazards assumption (Additional file 1: Text S1). Only age groups below 40 years (and to some extent comorbidities immunosuppression, obesity, and hypertension) did show slight deviations from proportional hazards; however, confidence intervals were wide and overlapped. When looking at model fits, all models fitted very well in the first 10 days after the positive test and again after 40 days, but slightly overestimated survival between 10 and 40 days if compared to the Kaplan-Meier curve based on the observed data (Additional file 1: Text S1).

## Discussion

### Principal findings

This nationwide analysis of almost 23,000 people who were hospitalized with COVID-19 in Switzerland found that mortality was around 40% higher in the first than in the second epidemic wave. We found that survival among patients hospitalized with COVID-19 started to decrease when ICU occupancy was around 70% or more. Our study confirmed the higher risk of death in men than in women and older than younger people. At younger ages, having any comorbid condition was more strongly associated with poor survival than at older ages. Cancer, chronic respiratory disease, chronic kidney disease, and cardiovascular disease were most strongly associated with poorer survival. We found little evidence for poorer survival in patients with hypertension or obesity as single comorbid conditions.

### Strengths and weaknesses of this study

Our study uses data from mandatory surveillance for the whole of Switzerland and includes around 92% of all people with laboratory-confirmed COVID-19 who were admitted to any hospital during the first two waves of the epidemic. The use of Bayesian survival models allowed us to fit complex models that included many interactions between variables. For example, we could examine both single comorbid conditions and the interactions between comorbidities.

There are weaknesses in the study that result from the data collection by the national surveillance systems. First, we excluded some patients due to missing information, mainly on comorbid conditions. Patient characteristics between those with and without missing comorbid conditions were similar, but comorbid conditions were mainly missing in patients who survived. Patients with missing information about comorbid conditions might more likely be free of comorbidity, leading to underestimating the association between comorbidity status and mortality. However, the potential bias is likely small since the proportion with missing data was relatively low (<8%). Second, information on obesity and chronic kidney disease as comorbidities was not recorded until mid-April 2020, and data on the number of ICU beds was not collected until mid-March 2020. We, therefore, restricted analyses of ICU occupancy and types of comorbidities to the second wave. Given the improvement in survival over time, the analyses of the more recent data will reflect the current clinical management of COVID-19 more closely. Third, a substantial proportion of COVID-19 deaths in older people occurred without hospitalization and these deaths were not included in analyses. These deaths likely occurred mainly in retirement and long-term care institutions, but information about the place of death was incomplete. Our findings of factors associated with survival may not be applicable to people who died outside the hospital. Fourth, our study includes the period when the SARS-CoV-2 alpha variant of concern rapidly became dominant in Switzerland but ended before the delta variant became established [24]. These variants of concern contain mutations that confer increased pathogenicity, compared with the wild type SARS-CoV-2, which dominated until January 2021 [25]. Therefore, our findings might underestimate the number of people admitted to the hospital and dying from COVID-19 since mid-2021. Still, the associations with sex, age, and type of comorbidity are unlikely to have been affected.

### Comparison with other studies

Our study adds to evidence about the association between survival with COVID-19 and ICU occupancy. Higher mortality during periods of high ICU occupancy has been shown [21, 22]. In Belgium, mortality was 1.42 times higher for patients admitted to the ICU during high ICU occupancy, and in England, mortality was 1.2 times higher. These studies categorized ICU occupancy as a binary or ternary characteristic (“ICU overflow” versus “no ICU overflow” and “0–45% occupancy” versus “45–85% occupancy” versus “>85% occupancy”). The strength of our analysis was that we modeled the non-linear association between survival and ICU occupancy and identified a threshold at around 70% overall occupancy, after which survival decreases. While this threshold might be specific to Switzerland and the second epidemic wave, this approach could be applied more widely.

Our analysis of survival trends over time complements the results of studies from England and Switzerland [26, 27]. Similar to our results, the study from England showed an increase in survival over time in patients admitted with COVID-19 to an intermediate care unit (IMCU) or intensive care unit. In the Swiss study, the authors collected detailed data from COVID-19 patients in a subset of Swiss hospitals, including information on admission to an IMCU or ICU after hospitalization. They found that overall hospital survival improved over time, but that survival of patients in IMCUs and ICUs was worse during the second epidemic wave than during the first. Our finding of a lower survival rate when ICU occupancy exceeds 70% is consistent with these results.

The decrease in survival at older ages and in men is in line with results from other studies [9–12]. Our study also contributes data on the association between comorbid conditions and decreased survival, which has been addressed in other studies [7, 8, 14–18]. We showed that comorbidities had a stronger detrimental effect on survival at younger than at older ages. While there are substantial interactions between specific comorbid conditions and age, this was not observed for sex. Also, we found no evidence of an increase in mortality in patients with obesity as a single comorbid condition, in contrast to findings from another study [28]. Further, we found no increase in mortality in patients suffering from hypertension alone, confirming other studies from Switzerland [4, 5]. Systematic reviews of relevant studies found that hypertension was associated with increased mortality, but these studies did not report whether hypertension was the sole condition [29–31]. This could suggest that patients’ hypertension, as long as it is under control, is not associated with worse survival, while the combination of hypertension and cardiovascular disease could lead to a more severe course. We found other potential interactions between comorbidities in both directions, but many findings were based on a few events and require further investigation. To our knowledge, previous studies had not taken into account interactions between different comorbidities; they could only estimate their average effects.

### Interpretation and implications

The better survival during the second wave of COVID-19 in Switzerland, compared to the first, is consistent with improvements in the clinical management of people hospitalized with COVID-19. These improvements might reflect both increased clinical experience and specific treatments. For example, the use of systemic corticosteroids in patients with severe and critical COVID-19 increased after the publication of results from clinical trials and a recommendation from the World Health Organization in September 2020 [32–40]. The decrease in survival starting at approximately 70% overall ICU occupancy shows a potential impact of operational pressure on mortality in patients hospitalized with COVID-19. The estimated absolute differences in survival were small, with an average difference of only 3.3% in survival between the lowest and highest ICU occupancy. Nevertheless, this translates into an estimated 137 deaths during high ICU occupancy that might have been prevented if ICU occupancy had remained below 70%. The available data did not allow us to examine ICU occupancy at the hospital level. This aspect of our analysis is ecological in nature and complements and contextualizes the data on ICU mortality at the individual level [25, 26]. It examines the general pressures exerted on the Swiss hospital system. We looked at occupancy among all available certified and add-on beds. During the second epidemic wave, 70% of overall ICU occupancy corresponded to approximately 85% occupancy among certified ICU beds. As ICUs are organized differently in different countries, our results may not generalize to other health care systems. In addition, if the ICU capacity changes in Switzerland in the future, our results may no longer apply to future epidemic waves. Nevertheless, our results strongly suggest that prevention and control of SARS-CoV-2 transmission in the population are needed long before intensive care units are fully occupied.

Our results on the risk of death for comorbid conditions alone and in combination with age and gender should help clinicians better assess the prognosis of their patients. Of note, the increased risk of death in men could not be explained by interactions with comorbidities or age. The higher mortality in men compared to women could be due to a higher prevalence of other risk factors, such as smoking, unhealthy diet, or lack of physical activity, data that were unavailable for our analyses [41–44]. Men might also be admitted to the hospital at a more advanced stage of the disease [9]. Lastly, there might be a biological explanation related to gender differences in immune responses [43, 45].

## Conclusions

The survival of hospitalized COVID-19 patients has improved over time in Switzerland, possibly due to improved knowledge in the treatment of SARS-CoV-2 infections. Survival was relatively stable until national ICU occupancy reached about 70%, after which it began to deteriorate. Our results suggest that operational pressures develop well before ICU full occupancy and before triage becomes necessary. Therefore, one should be cautious about concluding that the situation is under control as long as there is still capacity in the intensive care units.

## Supplementary Information

**Additional file 1: Figure S1.** Flowchart of data inclusion and exclusion. **Figure S2.** ICU occupancy during the second epidemic wave in Switzerland. **Figure S3-S6.** Additional model outputs. **Table S1-2.** Additional data description. **Text S1.** Further description of the statistical approach.

## Data Availability

The data are accessible to researchers upon reasonable request for data sharing with the corresponding author. Requests for data need to be approved by the Swiss Federal Office of Public Health.

## Abbreviations

COVID-19: Coronavirus disease 19
SARS-CoV-2: Severe acute respiratory syndrome coronavirus 2
ICU: Intensive care unit
aHR: Adjusted hazard ratio
CrI: Credible interval
IMCU: Intermediate care unit
