# Supplementary material for: Survival among people hospitalized with COVID-19 in Switzerland: a nationwide population-based analysis
Source: BMC Med. 2022 Apr 26;20:164. doi: 10.1186/s12916-022-02364-7 (PMC9038218; doi:10.1186/s12916-022-02364-7)

Supplementary materials to Anderegg et al.:

Survival among people hospitalized with covid-19 in Switzerland: a nationwide population-based analysis

Supplementary Figure S1. Flowchart of data inclusion and exclusion in study. To compare the two epidemic waves, all hospitalised covid-19 cases tested positive between February 24, 2020 and March 01, 2021 with complete covariate information were included. To compare survival by ICU occupancy and survival by comorbidity type data was restricted to the second epidemic wave (June 06, 2020 to March 01, 2021).

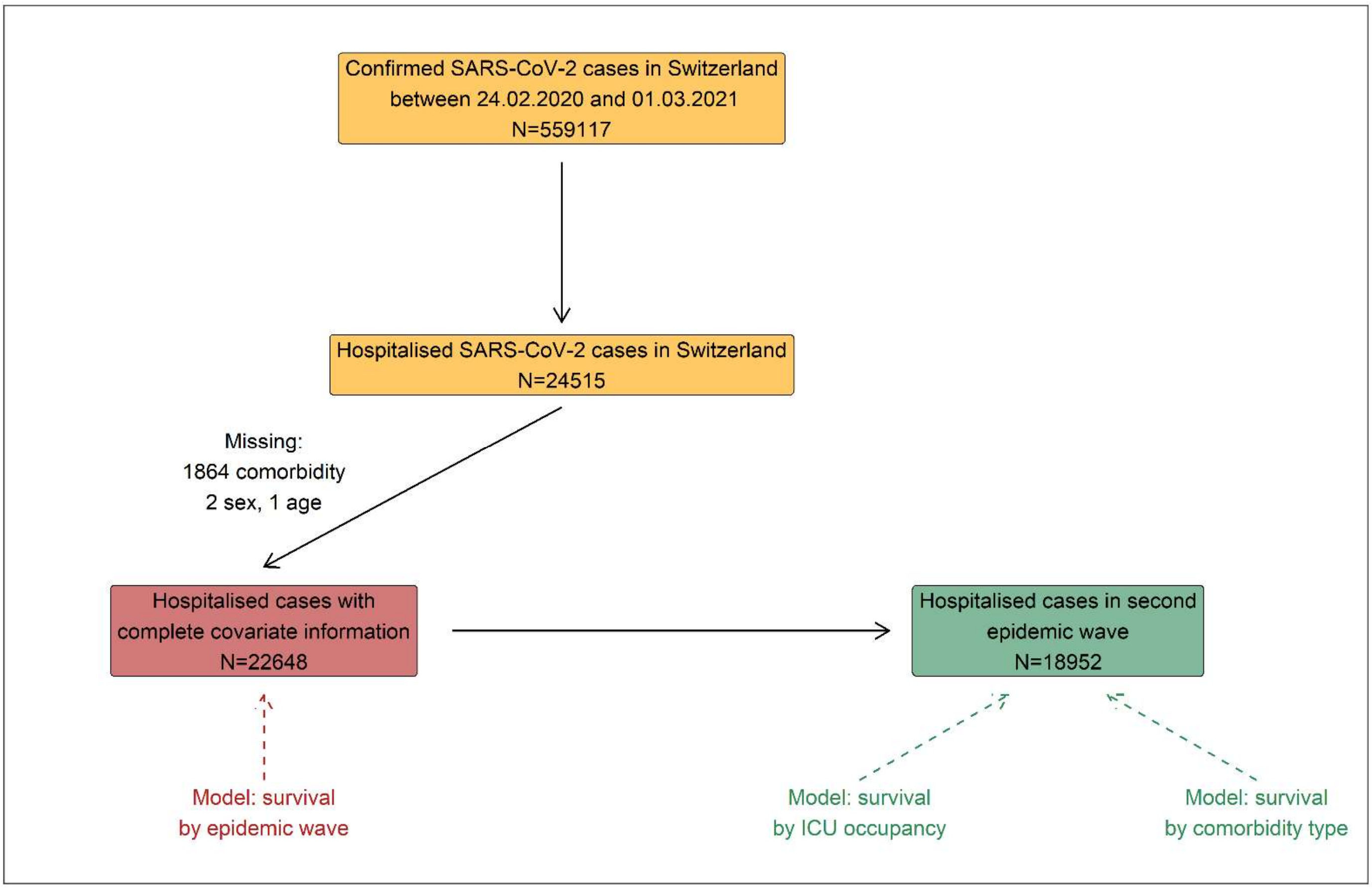

Supplementary Figure S2. ICU occupancy during the second epidemic wave (June 06, 2020 to March 01, 2021) in Switzerland. ICU occupancy over time (upper panel) as used in analyses, defined as total beds occupied (covid-19 and non-covid-19) at a day among all available ICU beds (certified and add-on beds) at that day. Number of total available ICU beds composed into certified and add-on beds over time (lower left panel). And the relationship between ICU occupancy (all beds) as used in analyses and ICU occupancy among certified beds (defined as total occupied beds among certified beds only).

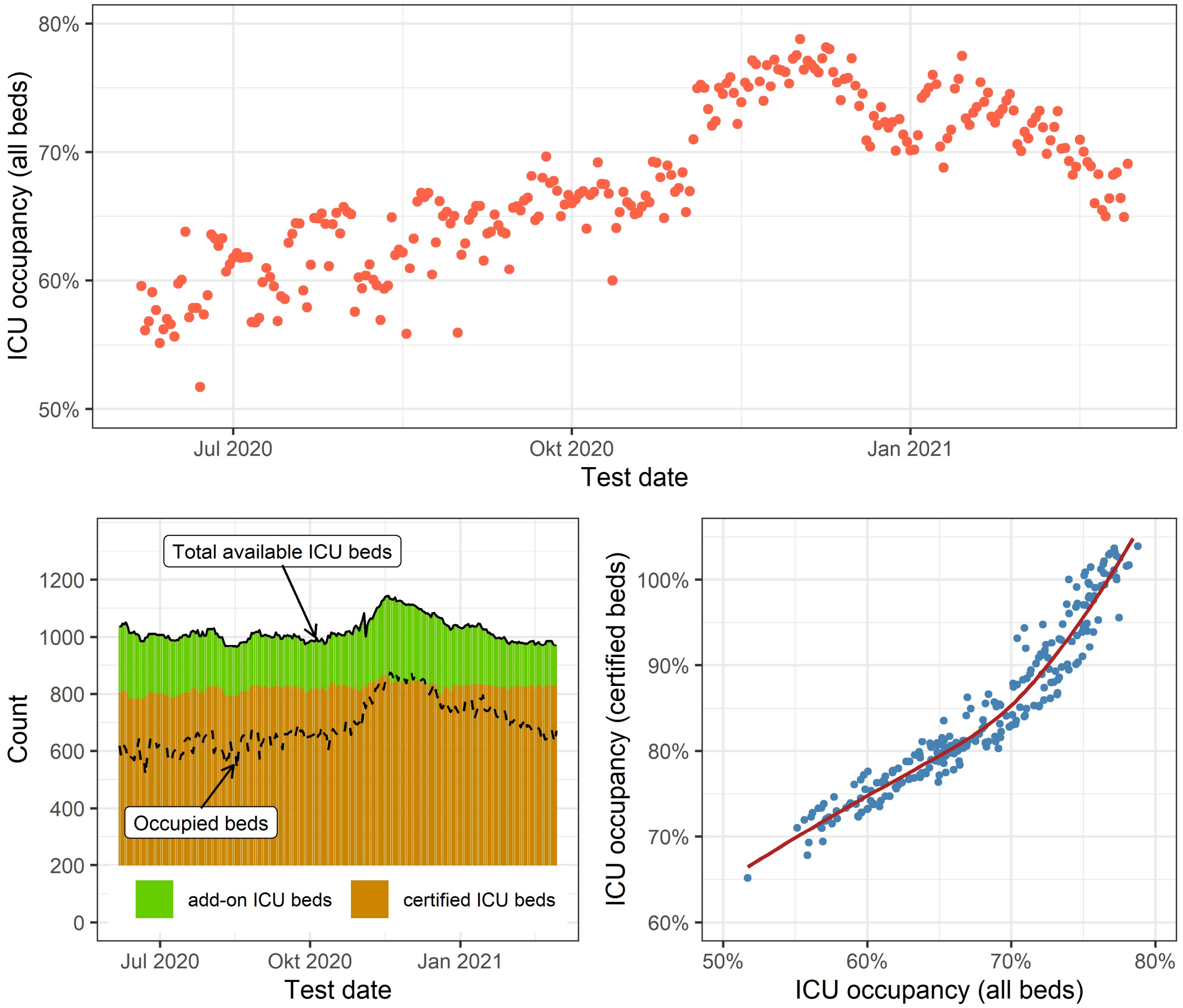

Supplementary Figure S3. Estimated baseline hazard rates with 95% credible interavls (CrI) (upper panel) and corresponding standardised predicted survival and 95% CrI over time by epidemic wave (lower panel). Results from models fitted to hospitalised covid-19 patients in both epidemic waves. The main model included covariates epidemic wave, sex, age, comorbidity status, and all two-way interactions between age, sex, and comorbidity status. The additional model had the same model structure, but including the number of comorbidities (0, 1, 2, 3+) instead of the comorbidity status. Standardisation (in the lower panel) is done by averaging daily predictions for the whole population by epidemic wave in each of the posterior samples (400 draws).

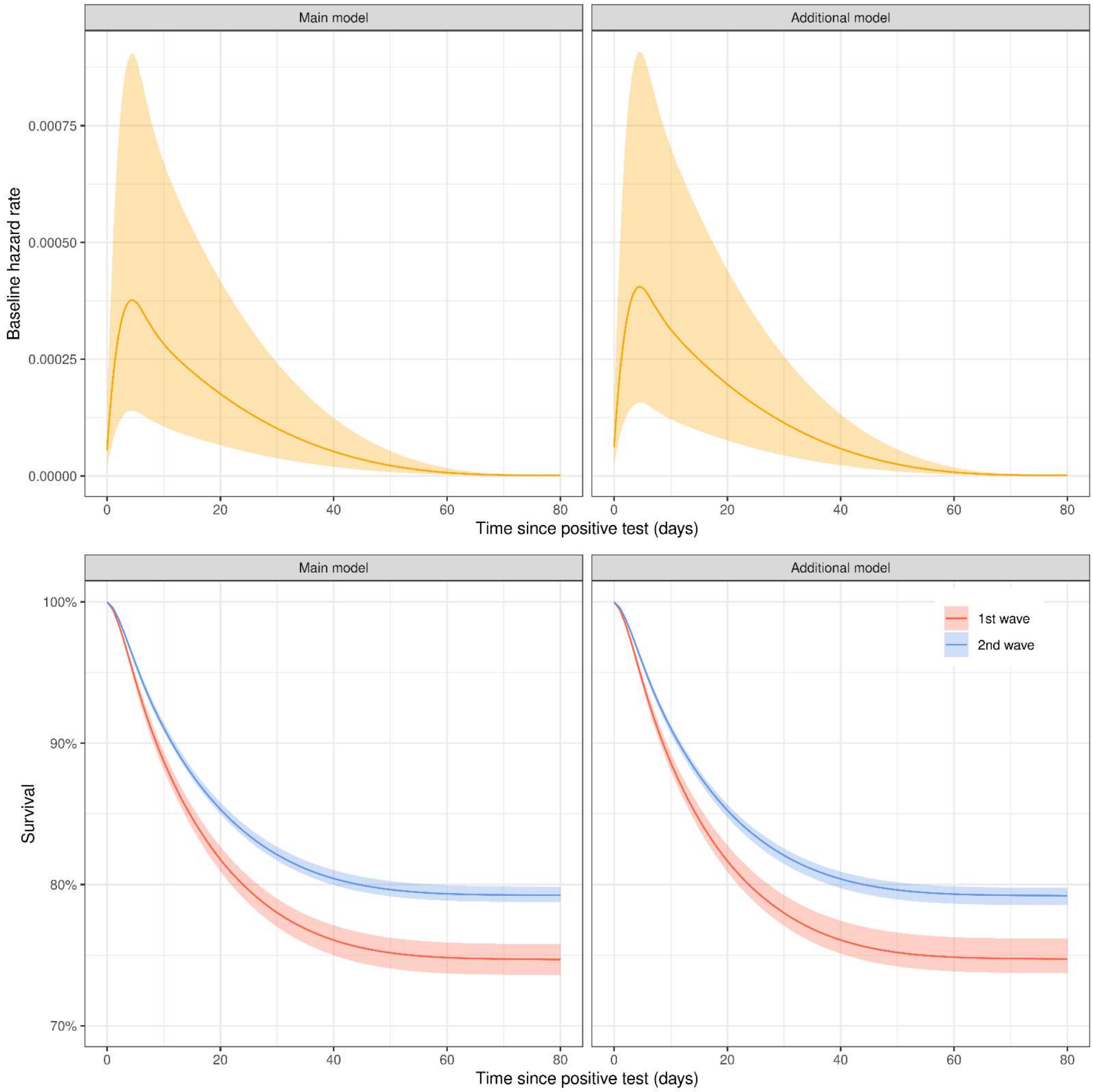

Supplementary Figure S4. Standardised predicted survival at 40 days after positive SARS-CoV-2 test among hospitalized covid-19 patients, by sex, age and number of comorbidities. Results from the model fitted to data from both epidemic waves including covariates epidemic wave, sex, age, number of comorbidites, and all two-way interactions between age, sex, and number of comorbidities. Standardisation is done by averaging first and second epidemic wave predictions of the whole population at day 40 day in each of the posterior samples (1000 draws).

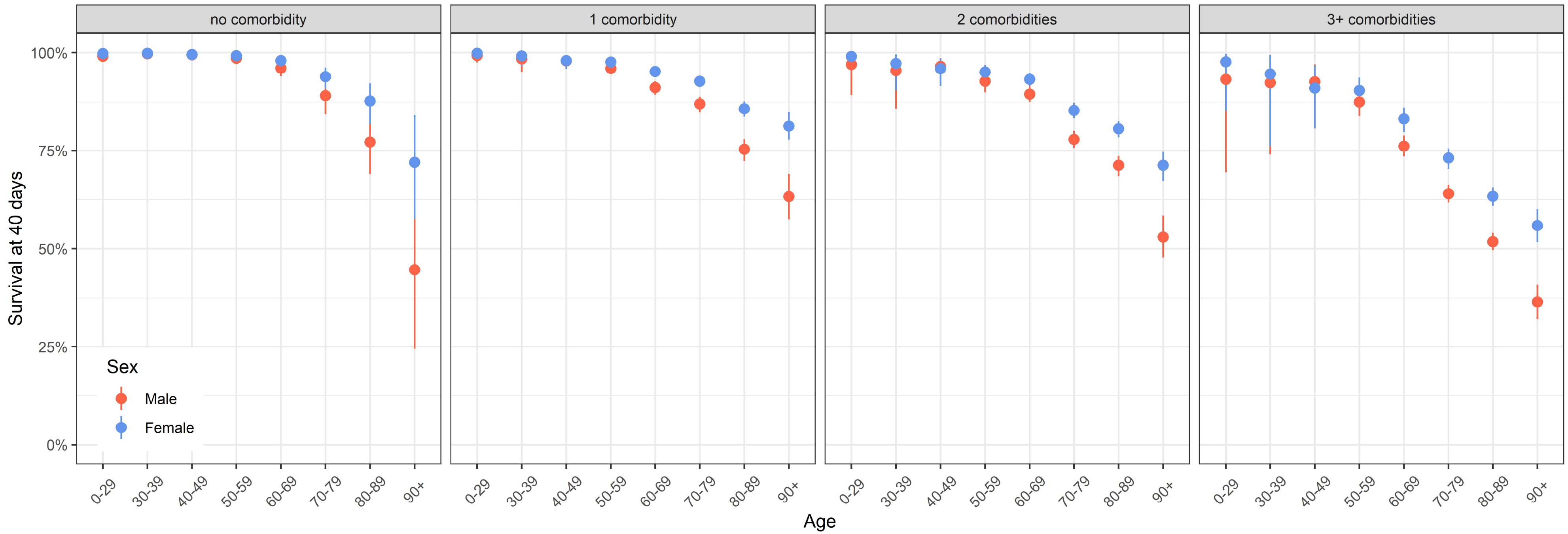

Supplementary Figure S5. Survival of hospitalised covid-19 patients by ICU occupancy. Adjusted hazard ratio of death by ICU occupancy relative to the baseline of 50% occupancy. Areas that are more transparent correspond to 95% credible intervals, less transparent ones to 50% credible intervals. Results from the model fitted to data from the second epidemic wave, including covariates ICU occupancy (modelled by restricted cubic splines with 3 knots), sex, age, comorbidity status, and the interaction between age and comorbidity status.

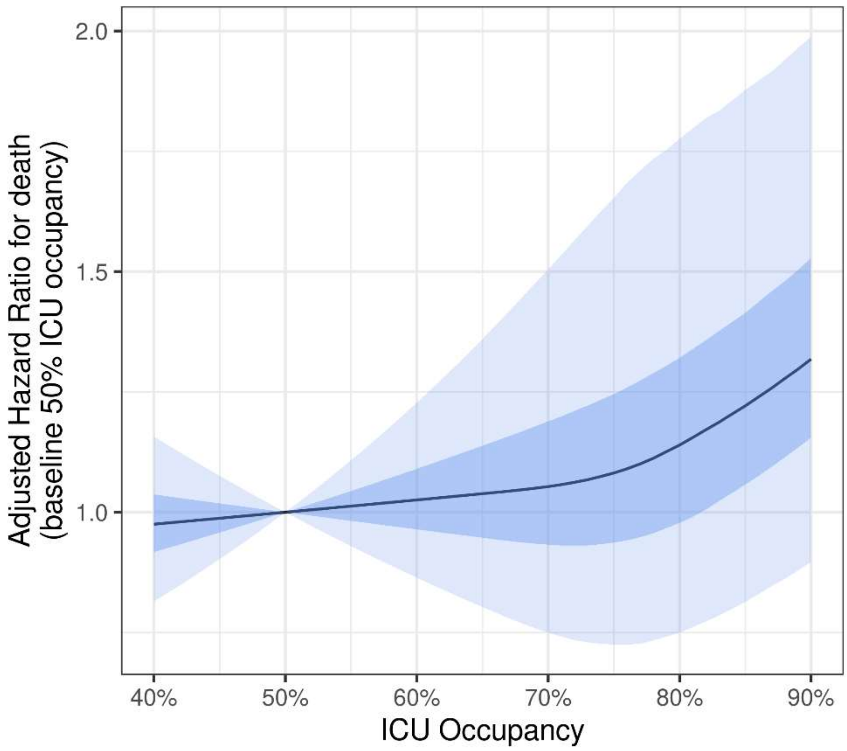

Supplementary Figure S6. Estimated adjusted hazard ratios (aHR) of death and 95% credible intervals for two-way interactions between individual comorbidity types. Estimates above 1 correspond to situations where the effect of both comorbidities together on mortality is larger than the sum of their individual effect (synergistic effect). Estimates below 1 correspond to situations where the effect of both comorbidities is smaller than the sum of their individual effects (antagonist effect). Results from the model fitted to data from the second epidemic wave, including covariates sex, age, the different comorbidity types, and two-way interactions between comorbidity types, between age and comorbidity types, and between sex and comorbidity types.

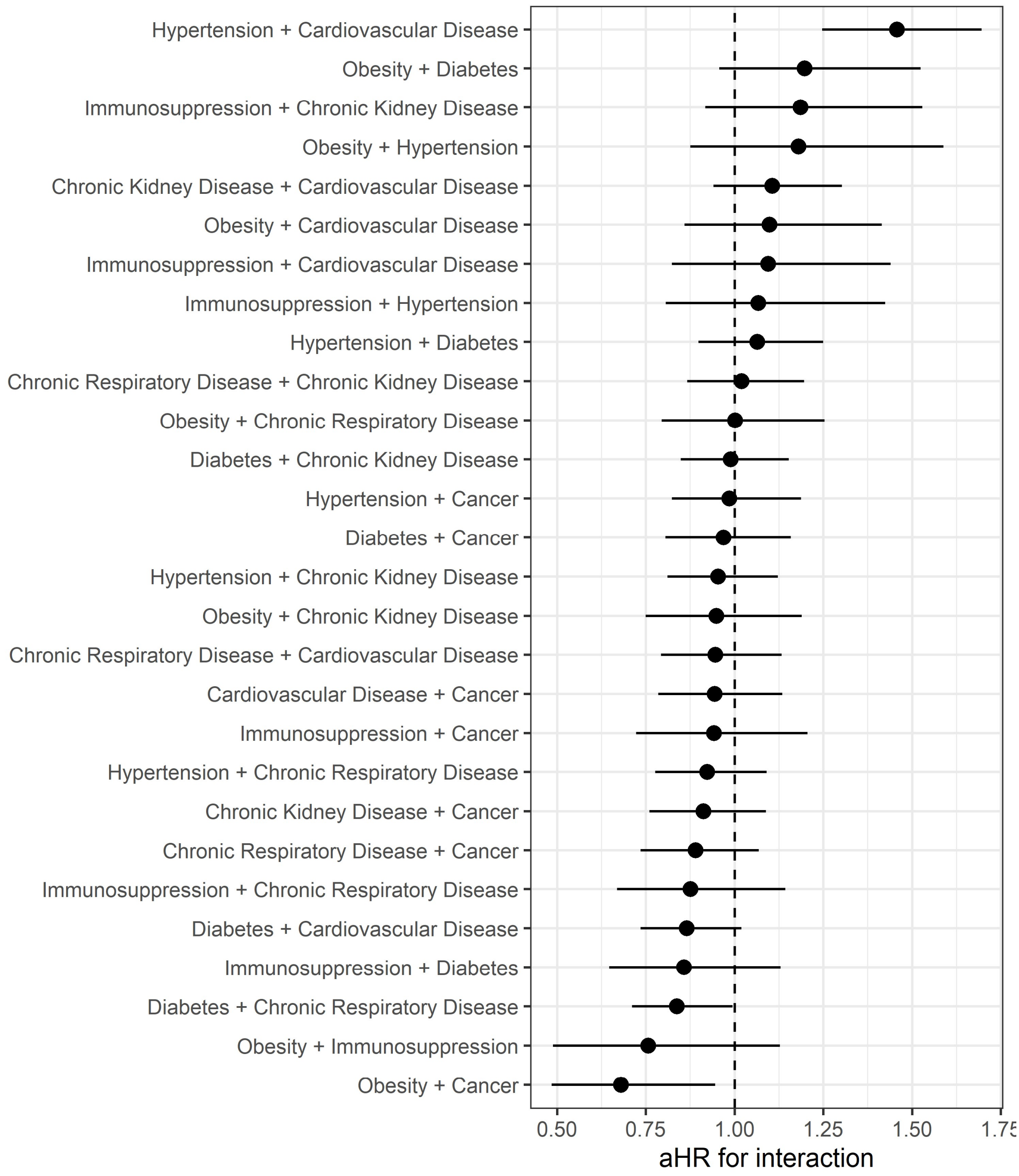

Supplementary Table S1. Comparison of deaths among confirmed SARS-CoV-2 cases hospitalised and non-hospitalised by epidemic wave. Deaths with missing comorbidity information are excluded (62 exclusions for hospitalised deaths, 234 for non-hospitalised deaths).

	**Deaths in 1st epidemic wave (n=1630)**		**Deaths in 2nd epidemic wave (n=7785)**	
	**Hospitalised deaths**	**Non-hospitalised deaths**	**Hospitalised deaths**	**Non-hospitalised deaths**
**Total**				
	897 (100%)	733 (100%)	3888 (100%)	3897 (100%)
**Sex**				
Female	292 (32.6%)	398 (54.3%)	1381 (35.5%)	2273 (58.3%)
Male	605 (67.4%)	335 (45.7%)	2507 (64.5%)	1624 (41.7%)
**Age [years]**				
Median (IQR)	81 (74-87)	88 (82-92)	82 (75-87)	88 (83-92)
0 - 29	1 (0.1%)	0 (0.0%)	3 (0.1%)	1 (0.0%)
30 - 39	4 (0.4%)	1 (0.1%)	4 (0.1%)	0 (0.0%)
40 - 49	4 (0.4%)	1 (0.1%)	22 (0.6%)	5 (0.1%)
50 - 59	31 (3.5%)	11 (1.5%)	98 (2.5%)	22 (0.6%)
60 - 69	94 (10.5%)	24 (3.3%)	382 (9.8%)	84 (2.2%)
70 - 79	252 (28.1%)	88 (12.0%)	1107 (28.5%)	444 (11.4%)
80 - 89	371 (41.4%)	321 (43.8%)	1666 (42.8%)	1679 (43.1%)
90+	140 (15.6%)	287 (39.2%)	606 (15.6%)	1662 (42.6%)
**Comorbidities**				
**Presence of any comorbidity**				
No comorbidity	19 (2.1%)	12 (1.6%)	88 (2.3%)	59 (1.5%)
Any comorbidity	878 (97.9%)	721 (98.4%)	3800 (97.7%)	3838 (98.5%)
**Number of comorbidities**				
0	19 (2.1%)	12 (1.6%)	88 (2.3%)	59 (1.5%)
1	149 (16.6%)	200 (27.3%)	569 (14.6%)	920 (23.6%)
2	254 (28.3%)	227 (31.0%)	901 (23.2%)	1174 (30.1%)
3+	475 (53.0%)	294 (40.1%)	2330 (59.9%)	1744 (44.8%)
**Presence of specific comorbidities[&]**				
Cancer	182 (20.3%)	67 (9.1%)	795 (20.4%)	303 (7.8%)
Cardiovascular disease	534 (59.5%)	390 (53.2%)	2561 (65.9%)	2306 (59.2%)
Chronic kidney disease	61 (6.8%)	57 (7.8%)	1437 (37.0%)	1034 (26.5%)
Chronic respiratory disease	209 (23.3%)	97 (13.2%)	960 (24.7%)	550 (14.1%)
Diabetes	258 (28.8%)	159 (21.7%)	1274 (32.8%)	805 (20.7%)
Hypertension	602 (67.1%)	426 (58.1%)	2551 (65.6%)	2190 (56.2%)
Immunosuppression	63 (7.0%)	22 (3.0%)	322 (8.3%)	68 (1.7%)
Obesity	8 (0.9%)	5 (0.7%)	430 (11.1%)	221 (5.7%)
Other	481 (53.6%)	419 (57.2%)	1254 (32.3%)	2079 (53.3%)

[&]Percentages can add up to more than 100% as patients may have multiple comorbidities.

Supplementary Table S2. Comparison of patients hospitalised with covid-19 with and without missing information about comorbid conditions.

	Comorbidity information	
	**Missing**	**Complete**
**Total**		
	1865 (100%)	22648 (100%)
**Sex**		
Female	876 (47.0%)	9555 (42.2%)
Male	988 (53.0%)	13093 (57.8%)
**Age [years]**		
0 - 29	141 (7.6%)	654 (2.9%)
30 - 39	110 (5.9%)	521 (2.3%)
40 - 49	201 (10.8%)	1076 (4.8%)
50 - 59	322 (17.3%)	2514 (11.1%)
60 - 69	327 (17.5%)	3898 (17.2%)
70 - 79	352 (18.9%)	5896 (26.0%)
80 - 89	312 (16.7%)	6266 (27.7%)
90+	99 (5.3%)	1823 (8.0%)
**Outcome**		
dead	62 (3.3%)	4785 (21.1%)
alive	1802 (96.7%)	17863 (78.9%)

Supplementary Text S1. Information about Bayesian survival models fitted.

Model structures and priors

For this study, we fitted the four models as described (simplified) below:

1) stan_surv((follow_up_time,death_status) ~ epidemic_wave + (age + sex + comorbidity_status)^2)
2) stan_surv((follow_up_time, death_status) ~ epidemic_wave + (age + sex + comorbidity_number)^2)
3) stan_surv((follow_up_time, death_status) ~ rms::rcs(ICUoccupancy) + age + sex + comorbidity_status + age:comorbidity_status) with, rms::rcs(ICUoccupancy) having 3 knots at percentiles 0.1, 0.5, 0.9.
4) stan_surv((follow_up_time, death_status) ~ age + sex +
(comorbidity_cardiovascular + comorbidity_cancer + comorbity_hypertension + ….)^2 +
age: (comorbidity_cardiovascular + comorbidity_cancer + comorbity_hypertension + ….) +
sex: (comorbidity_cardiovascular + comorbidity_cancer + comorbity_hypertension + ….))

We compared different formulations of model 1) to examine which of the two-way interactions were supported by the data. We compared model fits of different models with and without some of the two-way interactions by looking at the model fit's difference in expected log pointwise predicitive density (elpd_diff) (and its standard error se_diff) for a new dataset, estimated by approximate leave-one-out cross validation. Corresponding results are shown in the table below:

	Interactions included	Interactions excluded	elpd_diff	se_diff
Full model	+ age:sex<br>+ age:comorbidity_status<br>+ sex:comorbidity_status	None excluded	-2.6	2.7
Submodel A	+ age:sex<br>+ age:comorbidity status	- sex:comorbidity_status	-1.9	2.6
Submodel B	+ age:sex<br>+ sex:comorbidity_status	- age:comorbidity_status	-11.3	5.7
Submodel C	+ age:comorbidity_status<br>+ sex:comorbidity_status	- age:sex	-0.6	0.8
Submodel D	+ age:sex	- age:comorbidity_status<br>- sex:comorbidity_status	-10.5	5.7
Submodel E	+ age:comorbidity_status	- age:sex<br>- sex:comorbidity_status	0.0	0.0
Submodel F	+ sex:comorbidity_status	- age:sex<br>- age:comorbidity_status	-9.2	5.0
Submodel G	None included	- age:sex<br>- age:comorbidity_status<br>- sex:comorbidity_status	-8.3	5.1

The differences in elpd relative to their standard error were very small for all models that did include the interaction between age and comorbdity status (Full model, Submodel A, Submodel C, Submodel E). These models thus fitted the data equally well. Those excluding the interaction between age and comorbidity status (Submodel B, Submodel D, Submodel F, Submodel G) fitted worse. The best model fit was provided by Submodel E including only the interaction between age and comorbidity status. We thus decided to only include the age-comorbidity_status interaction for model 3) (and thus not include interactions between sex and age and between sex and comorbidity status). For model 4) we also did not include the interaction between sex and age, but - as we were looking not at the comorbidity status but at different individual comorbidities - we did include the interaction between individual comorbidities and sex next to the interaction between individual comorbidities and age.

For all models, we fitted cubic M-splines to model the baseline hazard and assumed the following weakly prior distribution for parameters:

- Intercept: Normal(location = 0, scale=20)
- Regression coefficients: Normal(location = 0, scale = 2.5)
- M-spline coefficients (for baseline hazard): Dirichlet(concentration = 1)

Model diagnostics: Proportional hazards assumption

Before fitting the models we checked for substantial deviations of the proportional hazards assumption by looking at log(-log(S(t)) versus t vor each categorical covariate, which should result in parallel lines. The plots are shown below. Only age groups<40 (and to some extent comorbidities immunosuppression, obesity and hypertension) did show slight deviations from the proportional hazards assumption, but as confidence intervals were large and overlapping, we felt that a proportional hazards models should provide meaningful results.

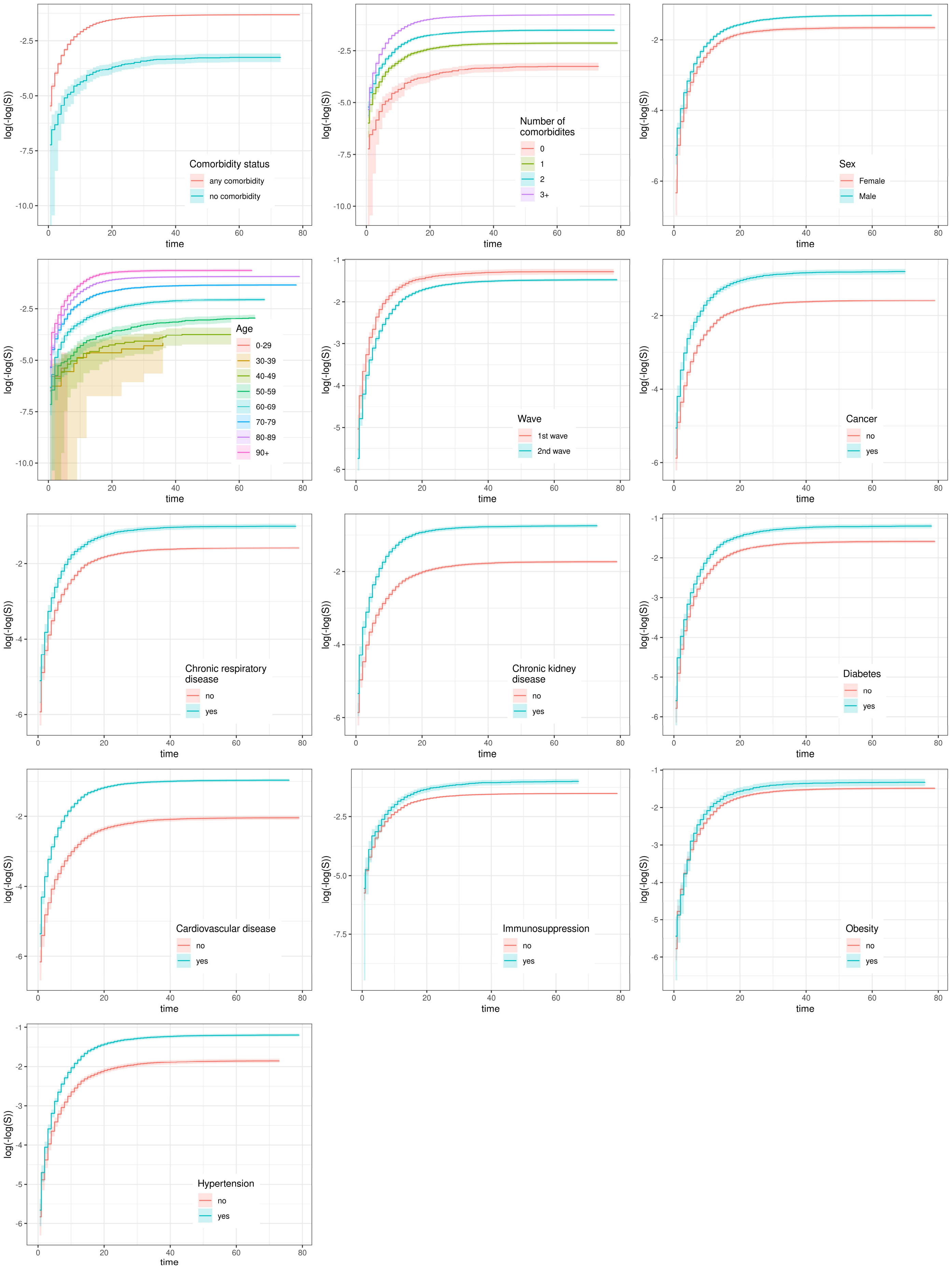

log(-log(S))
time
Comorbidity status
any comorbidity
no comorbidity
Number of comorbidites
0
1
2
3+
Sex
Female
Male
Age
0-29
30-39
40-49
50-59
60-69
70-79
80-89
90+
Wave
1st wave
2nd wave
Cancer
no
yes
Chronic respiratory disease
no
yes
Chronic kidney disease
no
yes
Diabetes
no
yes
Cardiovascular disease
no
yes
Immunosuppression
no
yes
Obesity
no
yes
Hypertension
no
yes

Model diagnostics: Check of model fits

To check model fits we used posterior predictive draws of the standardised survival probability (using all individuals in the estimation sample) and compared it to the observed Kaplan-Meier survival curve. The plots are shown below. In all cases, model fitted very well for times <10 and >=40 days, but somehow overestimated survival slightly inbetween.

Model epidemic waves

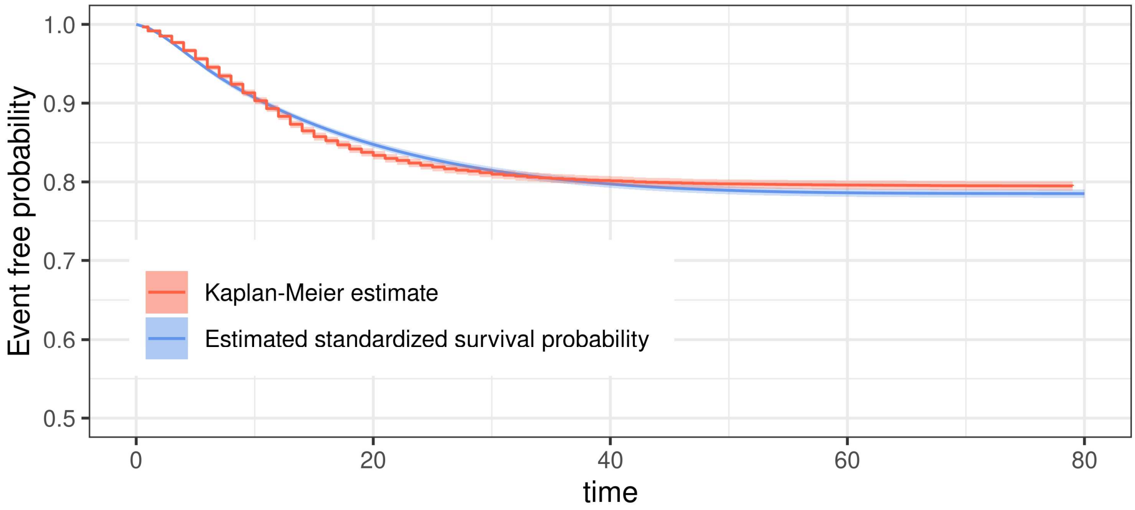

Model ICU capacity

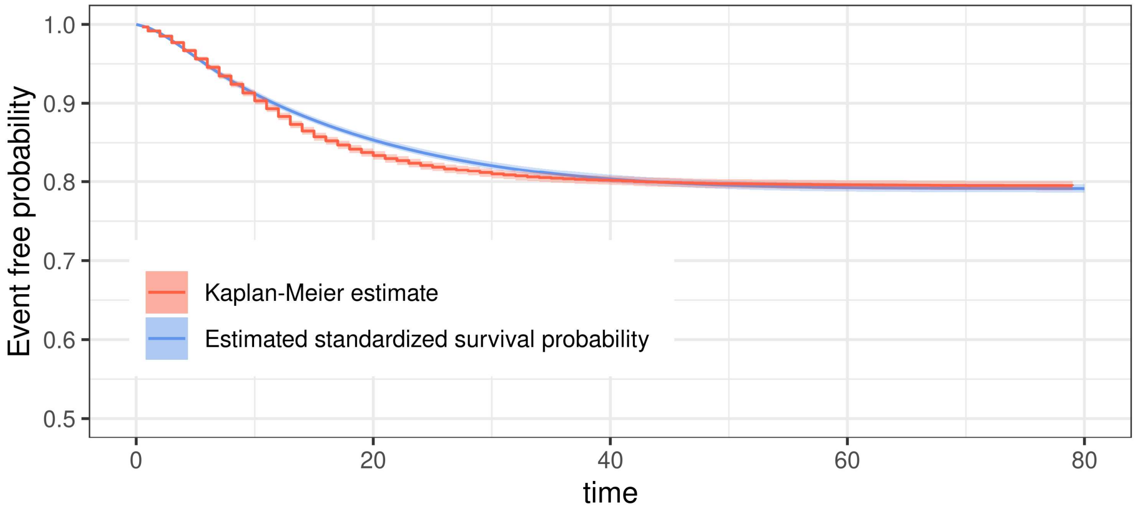

Model individual comorbidities

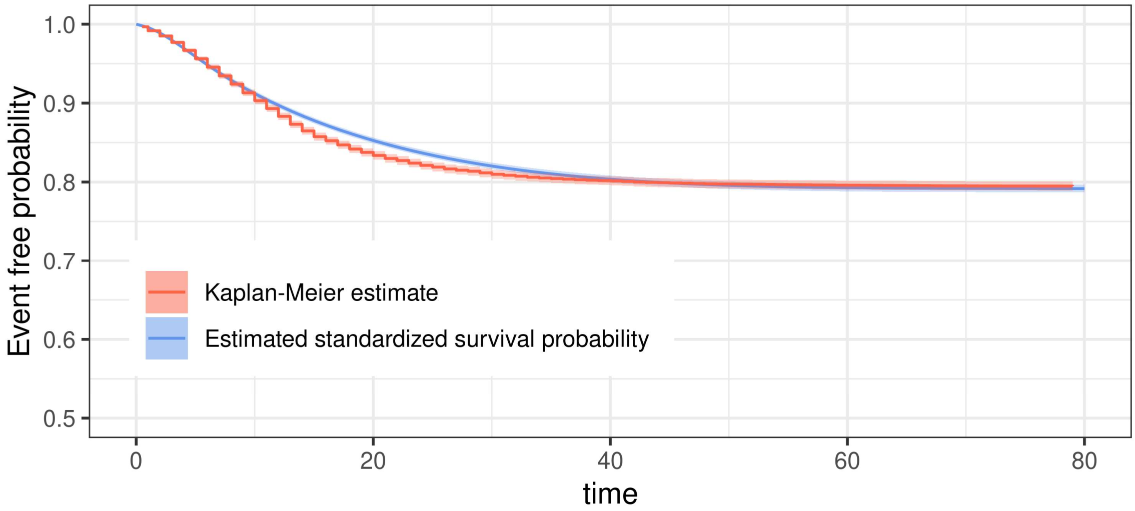

Supplement: Supplementary file 1 — Additional file 1: Figure S1. Flowchart of data inclusion and exclusion. Figure S2. ICU occupancy during the second epidemic wave in Switzerland. Figure S3-S6. Additional model outputs. Table S1-2. Additional data description. Text S1. Further description of the statistical approach. [file 12916_2022_2364_MOESM1_ESM.pdf]
